# Alcohol-Related Presentations to Emergency Departments on Days with Holidays, Social, and Sporting Events: An Integrative Literature Review

**DOI:** 10.1017/S1049023X23006507

**Published:** 2023-12

**Authors:** Stephanie Rae Hagan, Julia Crilly, Jamie Ranse

**Affiliations:** 1. School of Nursing and Midwifery, Griffith University, Gold Coast, Queensland, Australia; 2. Department of Emergency Medicine, Gold Coast Health, Gold Coast, Queensland, Australia; 3. Menzies Health Institute Queensland, Griffith University, Gold Coast, Queensland, Australia

**Keywords:** emergency nursing, emergency service, evidence-based emergency medicine, hospital, integrative review

## Abstract

**Introduction:**

Events, specifically those where excessive alcohol consumption is common, pose a risk to increase alcohol-related presentations to emergency departments (EDs). Limited evidence exists that synthesizes the impact from events on alcohol-related presentations to EDs.

**Study Objective:**

This integrative review aimed to synthesize the literature regarding the impact events have on alcohol-related presentations to EDs.

**Methods:**

An integrative literature review methodology was guided by the Preferred Reporting Items of Systematic Reviews and Meta-Analysis (PRISMA) Guidelines for data collection, and Whittemore and Knafl’s framework for data analysis. Information sources used to identify studies were MEDLINE, CINAHL, and EMBASE, last searched May 26, 2021.

**Results:**

In total, 23 articles describing 46 events met criteria for inclusion. There was a noted increase in alcohol-related presentations to EDs from 27 events, decrease from eight events, and no change from 25 events. Public holidays, music festivals, and sporting events resulted in the majority of increased alcohol-related presentations to EDs. Few articles focused on ED length-of-stay (LOS), treatment, and disposition.

**Conclusion:**

An increase in the consumption of alcohol from holiday, social, and sporting events pose the risk for an influx of presentations to EDs and as a result may negatively impact departmental flow. Further research examining health service outcomes is required that considers the impact of events from a local, national, and global perspective.

## Introduction

With many emergency departments (EDs) currently at or over capacity,^1^ understanding the impact of events on alcohol-related presentations to EDs can assist in health promotion and preventative strategies, future ED forecasting, ED planning, and ED resource allocation.^2^ Alcohol consumption has contributed to an increasing number of ED visits^3^ and a higher proportion of alcohol-related presentations to EDs are evident amongst males^4^ and occur overnight and on weekends.^5^ Furthermore, some people presenting to EDs with alcohol intoxication can have long stays in the ED^4^ and can be violent and/or aggressive.^5–8^

Alcohol, a central nervous system depressant, alters communication between the brain and body, exhibiting symptoms of poor concentration, slower reflexes, and increases the threat for risk-taking behaviour.^9^ The impact from the consumption of alcohol in many countries continues to grow^5,10,11^ with alcohol associated with many social and cultural events.^10^ It is not uncommon for alcohol to be served and/or consumed at some of these events, and in large quantities.^12^ With reports suggesting that binge drinking is attributed to 75% of all alcohol consumed^13^ and that 25% of people aged 14 and above consume more than four standard drinks in one sitting, at least monthly,^10^ continued cause for concern exists. This is especially so when considering the longer-term consequences of excessive alcohol consumption which increases the risk for preventable illness, disease, and adverse health effects such as: alcoholic liver cirrhosis, alcohol dependence, depression, suicide, cardiovascular disease, cancer, road traffic accidents, and assaults.^14^

Events that result in changes to alcohol intake volumes external to ED may result in additional patient presentations to ED and disrupt normal operational capacity. Such events may be considered in terms of being planned or unplanned. Examples of planned events include mass-gathering events (MGEs) such as music festivals and sporting events; policy events such as changes in alcohol legislation; and social events such as school leaver celebrations and certain public holidays, like New Year’s Eve.^2,15–17^ Examples of unplanned events include natural disasters such as earthquake, tsunamis, and hurricanes, humanitarian emergencies such as displaced people, and disease outbreaks such as Ebola, Severe Acute Respiratory Syndrome (SARS), and coronavirus disease 2019 (COVID-19).^18^

For some planned events, such as MGEs, there can be a recognized impact on local EDs, especially when alcohol is involved.^19–29^ Less is known regarding the impact unplanned events have on alcohol-related presentations to EDs. Today, events are occurring more frequently, posing the risk for an increase in alcohol-related presentations to EDs; however, the specific impact varied events have on alcohol-related presentations to EDs is unknown.

The overarching aim of this integrative literature review was to synthesize the literature regarding the impact events have on alcohol-related presentations to EDs. The research was guided by the following questions: How do events impact alcohol-related presentations to the ED? Are there certain periods where there has been an increase or decrease in these presentations? And how does this impact functioning of the ED?

## Methods

### Design

This integrative literature review was guided by the Preferred Reporting Items for Systematic Reviews and Meta-Analyses (PRISMA) Guidelines^30^ for data collection and Whittemore and Knafl’s framework^31^ for data analysis. An integrative review design was deemed appropriate as it aims to comprehensively synthesize the literature to generate new insights.

### Data Collection

Papers published from January 2012 through May 2021 were retrieved on May 26, 2021 from three sources: Medical Literature Analysis and Retrieval System Online (MEDLINE; US National Library of Medicine, National Institutes of Health; Bethesda, Maryland USA); Cumulative Index to Nursing and Allied Health Literature (CINAHL; EBSCO Information Services; Ipswich, Massachusetts USA); and Excerpta Medica Database (EMBASE; Elsevier; Amsterdam, Netherlands). The search strategy for MEDLINE included different combinations of Medical Subject Headings (MeSH) terms, CINAHL included subject headings and keywords that are relevant to the topic, and EMBASE used keywords by way of EmTree headings. All MeSH terms and keywords are outlined in Table 1. Search strings are presented in Supplementary Table S1, Table S2, and Table S3 (available online only).

**Table 1.** MeSH Terms/Key Words

	Alcohol	Emergency Department	Event
**MeSH Terms**	Alcoholic Intoxication Alcohol Abuse Alcohol Addiction Alcohol Dependence Alcoholism	Emergency Service, Hospital Hospital Emergency Service Emergency Medical Services Emergency Medicine Emergency Nursing	
**Keywords**	Alcohol Alcohol Drinking Alcohol Intoxication Alcohol Consumption Excessive Drinking Drunk Drinking Behavior	Emergency Department Emergency Room Accident and Emergency Casualty	Disruption Abnormal Interrupt Increase Decrease Surge Spike Rush Rise Fall Busy Quiet Extend Extra More Less

Abbreviation: MeSH, Medical Subject Headings.

The identified papers were screened for eligibility^30^ against inclusion and exclusion criteria, as outlined in Table 2. Covidence software^32^ was used to support the screening management of the systematic review. The software automatically removed duplicates. One reviewer independently screened 100% of title and abstracts (SH), whilst the other two reviewers independently screened 50% each (JC, JR). Moderation was resolved by the reviewer who did not screen initially. One reviewer (SH) examined 100% of the full-text articles, whilst the other two reviewers independently screened 50% each (JC, JR). Moderation was resolved by the reviewer who did not screen initially. Data were manually extracted and entered into tables in a Word (Microsoft Corp.; Redmond, Washington USA) document (SH). Extracted data were checked by another reviewer (JR). The main outcome of interest was a change in alcohol-related presentations to EDs from an event. Missing information not evident in the included articles when extracting data of interest were left blank in the tables.

**Table 2.** Inclusion and Exclusion Criteria

Inclusion	Exclusion
• Full-text article published in English • Peer-reviewed papers • Published between 2012 –2021 (ten years) • Papers relating to emergency department presentations of alcohol intoxication • Papers relating to events, such as: mass gatherings, social events, sporting events, public holidays, policy change • Papers discussing busy or quiet periods within the emergency department	• Theoretical papers • Discussion papers • Systematic reviews • Anonymous papers or authorship • Not evaluating the relationship between event and emergency department presentations of alcohol intoxication

### Data Analysis

Analysis was undertaken using the approach from Whittemore and Knafl’s framework.^31^ Information extracted from each paper included: a description of the event, inclusive of duration/dates; the impact of alcohol-related presentations to ED (increase, decrease, or no change); demographics, characteristics, and outcomes of alcohol-related presentations to ED with regards to the identified event; and a summary of the study characteristics including author(s), population, alcohol-related definitions, sample, and design.

## Results

**Figure 1.**
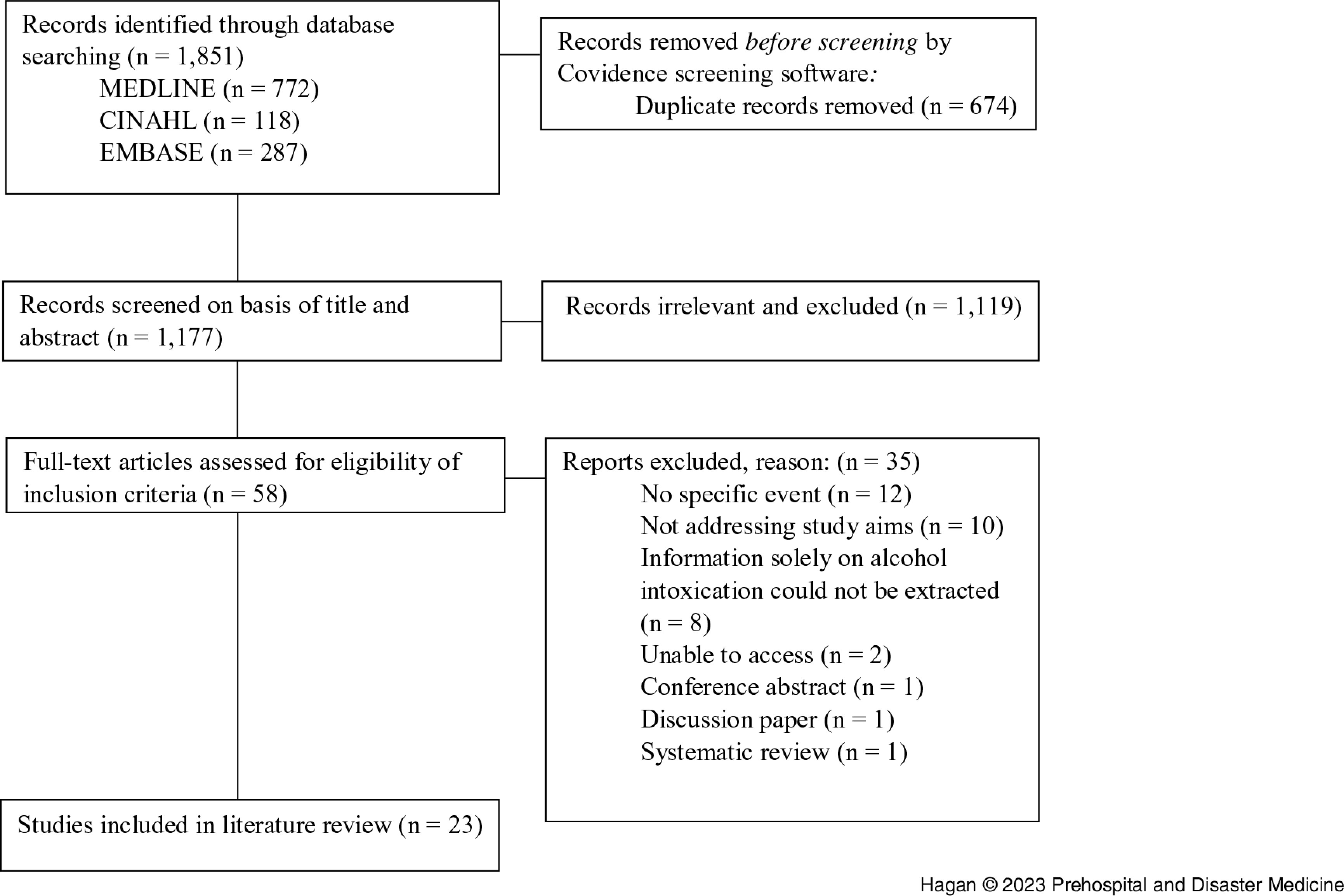
PRISMA^30^ Flow Diagram of Search Results.

A total of 23 papers met the criteria for inclusion (Figure 1), identifying 46 events that were grouped into six categories: (1) disasters: eg, earthquakes; (2) music festivals: eg, electronic dance music festivals; (3) policy changes: eg, changes in trading hours; (4) public holidays: eg, Christmas day; (5) social events: eg, birthday celebrations; and (6) sporting events: eg, Rugby World Cup. A summary of articles and study characteristics included in this integrative literature review is displayed in Table 3.

**Table 3.** Description of Study Characteristics

Study Characteristics				
Authors, Country of Origin	Population	Alcohol-Related Definition Used	Sample	Design
**Event Category:** ***Disaster***				
Kobayashi, et al^38^ Tokyo, Japan	6,395	Clinically diagnosed with AAI based on testimonies by patients or their companions. A blood ethanol level of ≥100mg/dL was used to support the diagnosis for those whose testimonies could not be elicited directly.	All AAI patients ≥ 20 years presenting to ED (April 2004-March 2007)	Retrospective time series study
**Event Category:** ***Music Festival***				
Chhabra, et al^22^ North America, USA	NS	Ethanol	18-29-year-olds presenting to a local ED from a three-day EDMF with ethanol and substance use (n = 28)	Descriptive retrospective case review
Ridpath, et al^23^ New York, USA	120,000	Alcohol use; positive hospital laboratory result	Cases of adverse events resulting in ED visits in patients ≥ 18 years old from a DMF in New York City (2013) (n = 22)	Retrospective observational cohort study
Ruest, et al^21^ USA	NS	Alcohol use; ICD-9 codes	13-30-year-olds presenting or transported to ED directly from music concerts (January 2011-December 2015) (n = 142)	Retrospective chart review
Stagelund, et al^24^ Roskilde, Denmark	10,630	Alcohol intoxication	Patients presenting to the MHCO at the 2012 Roskilde music festival who required referral to ED (n = 238)	Retrospective observational cohort study
**Event Category:** ***Policy Change***				
Atkinson, et al^44^ NSW, Australia	3.6 million	Alcohol consumption	Approximate population of New South Wales, Australia (2001-2021)	Agent-based simulation model
Castro-Marin, et al^25^ Arizona, USA	902,863	Narrative of alcohol consumption; lack of decision-making capacity by virtue of their apparent inebriation and its negative effect on judgment and mental status that’s further corroborated by elevated BACs, measured on arrival	All patients presenting to an ACS on the busiest day (Saturday) of a five-day professional golf tournament between 2012-2016	Retrospective observational cohort study
De Vocht, et al^33^ England	45,000	Alcohol; alcohol misuse	All emergency admissions to hospital for alcohol from 2010 to September 2014 (12 months post-closure)	Retrospective observational cohort study
Fierro-Fine, et al^43^ Iowa, USA	5,437	Ethanol intoxication ≥ 20mg/dL	Patients presenting to ED between 12:01am Saturday and 11:59pm Sunday from 2006-2014 with a first ethanol test result	Retrospective chart review using a multinomial logistical regression
Ford, et al^26^ Christchurch, New Zealand	NS	Alcohol consumption	Alcohol intoxication or alcohol-related presentations made by 14-87-year-olds (November 16-December 8, 2013) (n = 169)	Cross-sectional observational study
	NS	Alcohol consumption	Alcohol intoxication or alcohol-related presentations made by 14-87-year-olds (November 17-December 9, 2017) (n = 139)	
Fulde, et al^28^ Sydney, Australia	NS	Alcohol-related serious injuries; alcohol use documented in EDIS free text; voluntary information provided by the patient and enquiries by health care workers	All Category 1 and Category 2 patients presenting to ED in the 12 months before and 12 months after the 2014 changes to liquor licensing regulations (n = 564)	Blinded retrospective analysis
Gale, et al^39^ NSW, Australia	107,810	ICD-9-CM codes: 291; E860; 303; 305.0; 790.3; 980; V70.4 and ICD-10-AM codes: F10; R78.0; T51; X45; X65; Y15; Y90-91; Z04.0; Z72.1 or Z86.41	Acute alcohol presentations made by ≥ 15-year-olds to 39 EDs (1997-2011)	Age- and sex-specific time series analysis
Grigoletto, et al^27^ Italy	NS	Alcohol abuse; alcohol intoxication; blood alcohol level > 1.5g/L associated to functional impairment	Adolescents and young adults aged 13-24 years presenting to ED during the last three weeks of lockdown (April 10, 2020-May 3, 2020), first three weeks of reopening (May 4, 2020-May 27, 2020), and during the same periods in 2019 (n = 1,149)	Retrospective observational comparative analysis
Joseph, et al^34^ South India	NS	Alcohol consumption; alcohol intoxication; under the influence of alcohol	RTAs presenting to ED with AI and trauma – pre-ban (March 2017) (n = 194)	Retrospective observational cohort study
	NS	Alcohol consumption; alcohol intoxication; under the influence of alcohol	RTAs presenting to ED with AI and trauma – post-ban (April 2017) (n = 244)	
Kharasch, et al^29^ Boston, USA	NS	Acute alcohol intoxication or poisoning with signs or symptoms including ataxia; slurred speech; vomiting; disorientation; or alterations in consciousness by university personnel or calls for help by other students	Students transported by EMS to a local ED with signs or symptoms of alcohol intoxication two years prior and three years after the initiation of a university alcohol policy (n = 971)	Retrospective observational cohort study
Kisely, et al^40^ QLD, Australia	87,665	ICD-10; F10; T51.0; T51.9; and Z04.0	15-29-year-olds presenting to six EDs (April 2006-April 2009)	Interrupted time series analysis
Kisely & Lawrence, et al^41^ QLD, Australia	125,511	ICD-10; F10; Y90-91.9; R78.0; and Z04.0-0.5	15-29-year-olds: alcohol-related harms (April 2005-April 2010)	Interrupted time series analysis
	8,799	ICD-10; F10; Y90-91.9; R78.0; and Z04.0-0.5	15-29-year-olds: asthma or appendicitis (April 2005-April 2010)	
	9,709	ICD-10; F10; Y90-91.9; R78.0; and Z04.0-0.5	30-49-year-olds: alcohol-related harms (April 2005-April 2010)	
**Event Category:** ***Public Holiday***				
Griffin, et al^35^ Ireland	104,371	Alcohol consumption; alcohol intoxication	All ED presentations of self-harm and alcohol intoxication in Ireland from January 1, 2007 and December 31, 2015	Retrospective observational cohort study
Lloyd, et al^42^ Melbourne, Australia	52,197	ICD-10 coding of acute alcohol intoxication	Alcohol-related ambulance attendances (2000-2009)	Time series analysis
	27,990	ICD-10 coding of acute alcohol intoxication	ED alcohol intoxication presentations (2000-2009)	
	19,809	ICD-10 coding of acute alcohol intoxication	Hospital admissions of intoxication (2000-2009)	
**Event Category:** ***Social Event***				
Callaghan, et al^36^ Ontario, Canada	NS	ICD-10 codes: F10.0 – F10.9	All 12-30-year-old in-patient/ED admissions in Ontario, Canada (April 1, 2002-March 31, 2007)	Retrospective observational cohort study
Lloyd, et al^42^ Melbourne, Australia	52,197	ICD-10 coding of acute alcohol intoxication	Alcohol-related ambulance attendances (2000-2009)	Time series analysis
	27,990	ICD-10 coding of acute alcohol intoxication	ED Alcohol intoxication presentations	
	19,809	ICD-10 coding of acute alcohol intoxication	Hospital admissions of intoxication (2000-2009)	
**Event Category:** ***Sporting Event***				
Castro-Marin, et al^25^ Arizona, USA	902,863	Narrative of alcohol consumption; lack of decision-making capacity by virtue of their apparent inebriation and its negative effect on judgment and mental status that’s further corroborated by elevated BACs, measured on arrival	All patients presenting to an ACS on the busiest day (Saturday) of a five-day professional golf tournament between 2012-2016	Retrospective observational cohort study
Gardener, et al^20^ New Zealand	7,419	Alcohol consumption; alcohol intoxication	Presentations of alcohol intoxication to Auckland City Hospital during the 2011 Rugby World Cup	Retrospective observational cohort study
Noel, et al^19^ Marseille, France	NS	Alcohol consumption; alcohol intoxication; and ICD-10 codes	Alcohol intoxication presentations to three EDs in Marseille analyzed for three periods of pre, during, and post the EURO 2016 event	Retrospective observational cohort study
Miller, et al^37^ Victoria, Australia	16,940	Alcohol consumption; alcohol intoxication; alcohol misuse	Alcohol-related presentations to Geelong Hospital ED made by 13-96-year-olds (n = 9,494)	Retrospective cohort study
Lloyd, et al^42^ Melbourne, Australia	52,197	ICD-10 coding of acute alcohol intoxication	Alcohol-related ambulance attendances (2000-2009)	Time series analysis
	27,990	ICD-10 coding of acute alcohol intoxication	ED Alcohol intoxication presentations	
	19,809	ICD-10 coding of acute alcohol intoxication	Hospital admissions of intoxication (2000-2009)	

Note: Population as defined by study authors; sample refers to the cohort of patients included in the study.

Abbreviations: Acute Alcohol Intoxication (AAI); Alternate Care Site (ACS); Alcohol Intoxication (AI); Breath Alcohol Content (BAC); Emergency Department (ED); Emergency Department Information System (EDIS); Dance Music Festival (DMF); Electronic Dance Music Festival (EDMF); International Classification of Diseases (ICD); Medical Health Care Organization (MHCO); New South Wales (NSW); Queensland (QLD); Road Traffic Accident (RTA); United State of America (USA).

Studies included were undertaken in countries including Australia (n = 7), United States (n = 6), New Zealand (n = 2), Canada (n = 1), Denmark (n = 1), England (n = 1), France (n = 1), India (n = 1), Ireland (n = 1), Italy (n = 1), and Japan (n = 1). The design of studies varied, with the majority being retrospective observational cohort studies (n = 12)^19,20,23–25,27,29,33–37^ and time series analysis (n = 5).^38–42^ Of the 23 papers, there was only one identified disaster – earthquake.^38^ Four papers focused on music festivals,^21–24^ twelve focused on policy changes,^25–29,33,34,39–41,43,44^ two focused on public holidays,^35,42^ two on social events,^36,42^ and five focused on sporting events.^19,20,25,37,42^ Two papers were considered to fall into more than one category.^25,42^

Alcohol-related terms used were primarily reported as “alcohol intoxication” (n = 9)^19,20,24,27,29,34,35,37,42^ or “alcohol consumption” (n = 8).^19,20,25,26,34,35,37,44^ The terms “alcohol use” (n = 3),^21,23,28^ “alcohol misuse” (n = 2),^33,37^ “alcohol abuse” (n = 1),^27^ and “under the influence of alcohol” (n = 1)^34^ were also noted. Alcohol was also referred to as ethanol^22^ and ethanol intoxication >20mg/dL.^43^ Diagnosis codes related to alcohol were often used, and these included a range of International Classification of Diseases (ICD)-9 codes and ICD-10 codes (Table 3).

### Impact of Events on Alcohol-Related Presentations to EDs

An overview of the identified events and their impact on alcohol-related presentations to ED are outlined in Table 4. The impact was considered in terms of increase, decrease, or no change in alcohol-related presentations to the ED, as elaborated on below.

**Table 4.** Description of Event and Impact of Alcohol-Related Presentations to the ED

Publication Characteristics	Event Characteristics		Alcohol Presentations
	Event	Duration/Date(s)	
Event Category: *Disaster*			
Kobayashi, et al^38^	Earthquakes	500 days (April 4-March 17)	↓
Event Category: *Music Festival*			
Chhabra, et al^22^	Electronic Dance Music Festival	3 days	↑
Ridpath, et al^23^	Electronic Dance Music Festival	2 days (August 31-September 1, 2013)	↑
Ruest, et al^21^	115 Music Concerts	January 2011-December 2015	↑
Stagelund, et al^24^	2012 Roskilde Music Festival	10 days (June 30- July 9, 2012)	↑
Event Category: *Policy Change*			
Atkinson, et al^44^	Restriction of trading hours	2017 onwards	
	Extending trading to 12am		↑
	Extending trading hours to 2am		↑
	Extending trading hours to 11pm		↑
	1am closing time		↓
	3am closing time		↓
Castro-Marin, et al^25^	Alternate Care Site at event	2014 onwards	↓
De Vocht, et al^33^	Closure of a nightclub	September 2013 onwards	∼
	Closure of a restaurant and bar	November 2016 onwards	∼
	Local licensing guidance	2013-2014	∼
Fierro-Fine, et al^43^	University alcohol policy	2009 onwards	↑
Ford, et al^26^	Sale and Supply of Alcohol Act	December 2012 onwards	∼
Fulde, et al^28^	Changes to liquor licensing	February 24, 2014 onwards	↓
Gale, et al^39^	Increase in alcopops tax	April 27, 2008 onwards	↓
	Introduction of GST	July 1, 2000 onwards	↑
Grigoletto, et al^27^	COVID-19 lockdown	23 days (April 10-May 3, 2020)	↓
Joseph, et al^34^	Supreme Court Liquor Shop Ban	April 1, 2017 onwards	∼
Kharasch, et al^29^	University alcohol policy	2009 onwards	↑
Kisely, et al^40^	Increase in alcopops tax	April 28, 2008 onwards	∼
Kisely & Lawrence, et al^41^	Increase in alcopops tax	April 28, 2008 onwards	∼
Event Category: *Public Holiday*			
Griffin, et al^35^	August Bank Holiday	1 day (First Monday)	∼
	Christmas Day	1 day	↑
	Christmas Eve	1 day	↑
	Easter Monday	1 day	∼
	Easter Saturday	1 day	∼
	Easter Sunday	1 day	↑
	Good Friday	1 day	↓
	June Bank Holiday	1 day (First Monday)	↑
	May Bank Holiday	1 day (First Monday)	∼
	New Year’s Day	1 day	↑
	New Year’s Eve	1 day	↑
	October Bank Holiday	1 day (Last Monday)	∼
	St. Patrick’s Day	1 day (March 17)	↑
	St. Stephen’s Day	1 day (December 26)	↑
Lloyd, et al^42^	ANZAC	1 day (April 25)	↑
	Australia Day	1 day (January 26)	∼
	Boxing Day	1 day	∼
	Christmas Day	1 day	∼
	Easter/Good Friday	1 day	∼
	Labor Day	1 day (October 2)	∼
	New Year’s Day	1 day	↑
	Queen’s Birthday	1 day (June 12)	∼
	St. Patrick’s Day	1 day (March 17)	∼
Event Category: *Social Event*			
Callaghan, et al^36^	Hazardous birthday drinking	1,825 days (April 1, 2002-March 31, 2007)	↑
Lloyd, et al^42^	Last working day before Christmas	1 day	↑
Event Category: *Sporting Event*			
Castro-Marin, et al^25^	Professional Golf Tournament	5 days (2012-2016)	∼
Gardener, et al^20^	2011 Rugby World Cup	45 days (September 9- October 23, 2011)	↑
Noel, et al^19^	EURO-16 Football Cup	6 days (June 10-July 10, 2016)	↑
Miller, et al^37^	Geelong Football Games	36 x 1-day event (June 1 2005-February 16, 2010)	∼
Lloyd, et al^42^	AFL Grand Final	1 day (Last Saturday in September)	↑
	Commonwealth Games	12 days (March 15-26, 2006)	↑
	Formula 1 Grand Prix	1 day (Saturday early March/April 2006)	∼
	International soccer matches	1 day	∼
	Melbourne Cup Day	1 day (First Tuesday in November)	↑
	World Cup Soccer matches	1 day	∼

Note: ↑ = Increase; ↓ = Decrease; ∼ = No Change.

Abbreviations: Emergency Department (ED); Australian Football League (AFL); Australian New Zealand Army Corps (ANZAC); Goods and Services Tax (GST).

### Increase in Alcohol-Related Presentations to EDs

Twenty-seven of the 46 identified events impacted the ED by way of an increase in alcohol-related presentations.^19–24,29,35,36,39,42–44^ Four papers reported an increase in alcohol-related presentations following a music festival, particularly electronic dance music festivals, Roskilde music festival, and music concerts.^21–24^ Four papers reported an increase in alcohol-related presentations following a policy change, such as the implementation of a university policy,^29,43^ introduction of a Goods and Services Tax (GST),^39^ and a change in trading hours.^44^ Public holidays were documented in two papers^35,42^ with an increased risk and/or increase in alcohol-related presentations noted on: Christmas Eve; Christmas Day; New Year’s Eve; New Year’s Day; Easter Sunday; June Bank Holiday; St. Patrick’s Day; St. Stephen’s Day; the day prior to Australian New Zealand Army Corps (ANZAC) day; and the day prior to New Year’s Day.^35,42^ Two papers focusing on social events reported that birthday celebrations, especially ages 19, 20, 21, and 22,^36^ and the last working day before Christmas^42^ were associated with an increase in alcohol-related ED presentations.

Three papers focusing on sporting events reported an increase in alcohol-related presentations, most notably from the EURO-16 Football Cup, 2011 Rugby World Cup, Australian Football League (AFL) grand final day, Commonwealth Games and Melbourne Cup Day sporting events,^19,20,42^ and especially on game days as opposed to non-game days.^19^

### Decrease in Alcohol-Related Presentations to EDs

Seven papers reported on eight events that impacted the ED by way of a decrease in alcohol-related presentations.^25,27,28,35,38,39,44^ Five papers reported a decrease in alcohol-related presentations following policy changes.^25,27,28,38,44^ Good Friday was the only documented public holiday where a decrease in alcohol-related presentations was reported.^35^

### No Change in Alcohol-Related Presentations to EDs

Nine papers reported no evident change in alcohol-related ED presentations across 24 events.^25,26,33–35,37,40–42^ Five papers identified no change in presentations following policy changes pertaining to a change in trading hours, increase in alcopops tax, and the implementation of new licensing laws.^26,33,34,40,41^ One paper identified no change in presentations from public holidays including August, May, and October bank holidays, and Easter Monday and Saturday.^35^ Two papers identified no change from sporting events including professional golf tournament and Geelong Football games.^25,37^ One paper focusing on both public holidays and sporting events reported no change in presentations from the following events: Australia Day; Boxing Day; Christmas Day; Easter; Labor Day; Queen’s birthday; St. Patrick’s Day; Formula 1 Grand Prix; International Soccer matches; and World Cup Soccer matches.^42^

### Characteristics, Demographics, and Outcomes of Alcohol-Related Presentations to EDs from Events

Of the included studies, a summary of the characteristics, demographics, and outcomes of alcohol-related ED presentations from events is presented in Supplementary Table S4 (available online only). Nine papers reported on the age of patients with ages ranging from 12 to >65 years.^19,21–23,27,29,36,38,43^ Where sex (or gender) was included, men were reported as more likely to present in seven studies;^19,22,27,36,38,39,43^ women were more likely to present in two studies.^21,23^ Four papers included breath or alcohol levels ranging from 104mg/dL to 412mg/dL^21,27,29,43^ and one paper elaborated on other substances such as amphetamines and marijuana used in combination with alcohol.^22^ Eight papers identified outside normal working hours and weekends as busy periods in EDs, specifying times from 5:00pm to 02:15am are considered “high alcohol times,” and individual days such as opening ceremonies for sporting events.^20,22,27,28,35,36,38,43^ The ED length-of-stay (LOS) was only reported in three United States studies where the events were one music festival,^22^ a range of music festivals,^21^ and a university alcohol policy.^29^ For these studies, ED LOS was noted to be approximately 4.5 hours: median 265 minutes;^22^ mean 250 minutes;^21^ and mean 253 minutes.^29^

## Discussion

This integrative review of the literature explored the impact events have on alcohol-related presentations to EDs. The rate of alcohol-related presentations to EDs was not clearly articulated in the studies included in this review. Key findings that emerged from the review indicate that for some events, such as music festivals, public holidays, social events, and large sporting events, there was an increase in alcohol-related presentations to EDs. Impact tended to be reported in terms of number of presentations, ED LOS, and discharge disposition from ED. For those events where there was no change in alcohol-related presentations, this may reflect the nature of the event, availability of medical services at the event, or that there was an increase in ED presentations from the event, but they were not necessarily alcohol-related or given an ICD-10 code of alcohol intoxication due to other illness and accompanying injuries. In order to support and plan for future events and impacts on EDs from people who are intoxicated from alcohol, the following discussion is framed around considerations of clinical, workforce, and policy strategies.

### Clinical Strategies

Strategic planning for the impact events may have on EDs should have set goals and outcomes and be guided by prior evidence.^45^ This review found that certain types of events, including music festivals with crowds of >5000, public holidays, especially Christmas Eve, and large sporting events such as the EURO-16 Football Cup, impacted EDs more so than others in terms of alcohol-related presentations. Clinically driven strategies such as in-event health services^2^ and community sobering shelters have been reported to reduce ED presentations of alcohol intoxication,^25^ and thus warrant consideration during and following these types of planned events.

### Workforce Strategies

With a noted increase in ED presentations from particular events, appropriate staffing is needed to meet patient demands.^45,46^ Such resourcing considerations for the ED include staff skill mix; patient acuity; departmental flow of other patients; and the individual needs of the patient.^45,46^ Along with medical and nursing workforce, broader specialist workforce support from social workers and Alcohol and Other Drugs Services (AODS) warrant consideration. The ability to “flex up” the number of these staff and extend usual working hours may be required and should be included in known event planning considerations. Further research regarding the economic benefits of having additional staff at the event versus additional staff in the ED should be explored.

### Policy and Education Strategies

When public policy changes were considered as an event, studies included in this review reported either an increase, reduction, or no change in ED impact regarding alcohol-related presentations. Alcohol-related policy measures range from changing liquor licensing opening hours,^28,44^ reducing the affordability of alcoholic products,^39–41,47^ and prohibiting the consumption of alcohol and/or possession of open containers in public spaces.^48^ Along with these policy measures, educational efforts to shift the cultural acceptance of alcohol use by advertising the adverse health effects of alcohol should be pursued, especially for the younger, more vulnerable population.^49^

## Limitations

This integrative review was focused on the impact events have on alcohol-related presentations to EDs. Events can impact EDs in other ways, such as trauma/injury presentations, however the focus of this review was on alcohol intoxication. This review purposefully focused on studies of events and the impact of alcohol-related, specifically intoxication, presentations to the ED. Other reviews have included broader alcohol-related impacts such as accident and injuries that were purposefully excluded here. Findings are limited to those nations for which papers were retrieved, and as such, countries such as low- and middle-income countries may not have been included in the review.

## Conclusion

This integrative review explored literature regarding the impact events have on alcohol-related ED presentations. With events grouped into six categories, EDs were most impacted by an increase in presentations from music festivals, public holidays, and in some cases, the day prior to public holidays and large sporting events. Disasters had little impact on alcohol-related presentations to the ED. The consumption of alcohol and binge drinking behaviors at events resulting in an ED presentation is cause for continued efforts to direct public health and emergency care strategies to prevent or minimize alcohol-related harm. Further research examining health service outcomes is required that considers the impact of events on EDs from a local, national, and global perspective.

## Supplementary material

For supplementary material accompanying this paper visit https://doi.org/10.1017/S1049023X23006507. click here to view supplementary material
