# Supplementary material for: Alcohol-Related Presentations to Emergency Departments on Days with Holidays, Social, and Sporting Events: An Integrative Literature Review
Source: Prehosp Disaster Med. 2023 Oct 25;38(6):764–73. doi: 10.1017/S1049023X23006507 (PMC10694469; doi:10.1017/S1049023X23006507)
Supplement: Supplementary file 1 [file S1049023X23006507sup001.docx]

**SUPPLEMENTARY TABLES**

**Supplementary Table 1.** MEDLINE search method

| **Search date:** 26/05/21 | |
| --- | --- |
| **Set** | **MESH terms** |
| 1 | alcoholic intoxication/ or alcoholism/ or binge drinking/ |
| 2 | exp alcohol drinking/ |
| 3 | Alcoholism/ |
| 4 | ((ETOH or alcohol*) adj3 (intox* or drink* or drunk* or excess* or consum* or binge*)).ti,ab. |
| 5 | (binge* adj2 drink*).ti,ab. |
| 6 | or/1-5 |
| 7 | Emergency Service, Hospital/ |
| 8 | Emergency Medical Services/ |
| 9 | emergency medicine/ |
| 10 | Emergency Nursing/ |
| 11 | (emergency adj2 (room? or unit? or department? or accident or service? or ward?)).ti,ab. |
| 12 | casualt*.ti,ab. |
| 13 | or/7-12 |
| 14 | 6 and 13 |
| 15 | (disrupt* or abnormal* or interupt* or increas* or decreas* or surge* or rush* or rise* or fall* or busy* or quiet or extend* or extra or more or less).ti,ab. |
| 16 | 6 and 13 |
| 17 | (disrupt* or abnormal* or interupt* or increas* or decreas* or surge* or rush* or rise* or fall* or busy* or quiet or extend* or extra or more or less).ti,ab. |
| 18 | 14 and 15 |
| 19 | limit 18 to (english language and yr="2012 -Current") |

**Supplementary Table 2.** CINAHL search method

| **Search date:** 26/05/21 | |
| --- | --- |
| **Set** | **Subject headings** |
| S1 | MH alcoholic intoxication OR alcoholism OR binge drinking |
| S2 | (MH "Alcoholic Intoxication") OR (MH "Alcoholism") OR (MH "Alcohol Abuse") |
| S3 | (MH "Binge Drinking") |
| S4 | (MH "Alcohol Drinking") |
| S5 | (MH "Alcoholism") |
| S6 | TI ( (ETOH or alcohol*) N2 (intox* or drink* or drunk* or excess* or consum* or binge*) ) OR AB ( (ETOH or alcohol*) N2 (intox* or drink* or drunk* or excess* or consum* or binge*) ) |
| S7 | TI binge* N2 drink* OR AB binge* N2 drink* |
| S8 | S1 OR S2 OR S3 OR S4 OR S5 OR S6 OR S7 |
| S9 | (MH "Emergency Service") |
| S10 | (MH "Emergency Medical Services") |
| S11 | (MH "Emergency Medicine") |
| S12 | (MH "Emergency Nursing") |
| S13 | TI ( emergency N2 (room or unit or department or accident or service or ward) ) OR AB (emergency N2 (room or unit or department or accident or service or ward) ) |
| S14 | TI casualt* OR AB casualt* |
| S15 | S9 OR S10 OR S11 OR S12 OR S13 OR S14 |
| S16 | TI ( disrupt* or abnormal* or interupt* or increas* or deceas* or surge* or rush* or rise* or fall* or busy* or quiet or extend* or extra or more or less ) OR AB ( disrupt* or abnormal* or interupt* or increas* or deceas* or surge* or rush* or rise* or fall* or busy* or quiet or extend* or extra or more or less ) |
| S17 | S8 AND S15 AND S16 |

^MH: indicates that a subject heading has been searched.^

**Supplementary Table 3.** EMBASE search method

| **Search date:** 26/05/21 | |
| --- | --- |
| **Set** | **Subject headings** |
| 1 | 'alcohol intoxication'/de OR 'binge drinking'/de OR 'drinking behavior'/de OR 'alcoholism' |
| 2 | ((etoh OR alcohol*) NEAR/2 (intox* OR drink* OR drunk* OR excess* OR consum* OR binge*)):ti,ab |
| 3 | (binge* NEAR/2 drink*):ti,ab |
| 4 | #1 OR #2 OR #3 |
| 5 | 'hospital emergency service'/de OR 'emergency health service'/de OR 'emergency medicine'/de OR 'emergency nursing'/de |
| 6 | (emergency NEAR/2 (room OR unit OR department OR accident OR service OR ward)):ti,ab |
| 7 | casualt*:ti,ab |
| 8 | #5 OR #6 OR #7 |
| 9 | disrupt*:ti,ab OR abnormal*:ti,ab OR interupt*:ti,ab OR increas*:ti,ab OR decreas*:ti,ab OR surge*:ti,ab OR rush*:ti,ab OR rise*:ti,ab OR fall*:ti,ab OR busy*:ti,ab OR quiet:ti,ab OR extend*:ti,ab OR extra:ti,ab OR more:ti,ab OR less:ti,ab |
| 10 | #4 AND #8 AND #9 |
| 11 | #10 AND 'Conference Abstract'/it |
| 12 | #10 NOT #11 |
| 13 | #12 AND [english]/lim AND [2012-2021]/py |
| 14 | #12 AND [english]/lim AND [2012-2021]/py AND [embase]/lim |

**Supplementary Table 4.** Characteristics, demographics and outcomes of alcohol-related presentations to EDs regarding events

| **Publication characteristics** | **Characteristics** | | | **Demographics** | | **Outcomes** | | | **Other outcomes** |
| --- | --- | --- | --- | --- | --- | --- | --- | --- | --- |
|  | **Event** | **Busy periods** | **Quiet periods** | **Age (years)** | **Gender dominance** | **LOS** | **Disposition** | **BAL** |  |
| **Event category: *Disaster*** | | | | | | | | | |
| Kobayashi et al., (37) | Earthquakes | 51.1% weekend presentations: (n=3268/6395)  β (95% CI):  0.08* (−0.01* to 0.14)  ↑ male presentations in Winter compared to Spring (p=0.01*)  ↑ female presentations on non-working days (p=0.02*)  ↑ presentations amongst older people in Winter  (p=<0.01*) | ↓ male presentations during daytime earthquakes only (p=0.03*)  β (95% CI):  0.19 (-0.36 to -0.02*) | Mean (SD):  42.6 (16.9) | 71.8% Male  (n=4592/6395) |  | ↓ ED volume during daytime earthquakes  (95% CI):  0.08* (−0.01* to 0.14) |  |  |
| **Event category: *Music Festival*** | | | | | | | | | |
| Chhabra et al, (22) | Electronic Dance Music Festival | 17:00 – 02:15am  (all presentations) | 13:00 – 17:00  (no presentations) | Median (IQR): 21 (19.5-24)  Range: 18-29 | 53.57% Male (n=15/28) | Median (IQR):  265 minutes (210-347)  Range:  66-660 minutes | ↑ 2 ward admissions  ↑ 1 ICU admission |  | 64.3% Ethanol (n=18)  46.4% Amphetamine (n=13)  28.6% Marijuana (n=8). |
| Ridpath et al., (23) | Electronic Dance Music Festival |  |  | Median: 21  Range: 16-29  ≥20 = 55% | 59% Female  (n=13/22) |  | ↑ 5 ICU admissions (23%)  ↑ 2 deaths (9%) |  |  |
| Ruest et al., (21) | 115 Music Concerts |  |  | Mean (SD): 19.5 (3.3)  <18 = 33% (n=47)  <21 = 72% (n=102) | 71% Female  (n=101) | Mean (SD): 4.3 hours (3.4) | ↑ 142 ED presentations: 90% treated and discharged home (n=128)  61% (n = 86) required clinical interventions  46% received IVF (n = 65) | Mean (SD):  242mg/dL (70)  (n=60) | 48 of 115 concerts caused an increase in presentations, and these were: 31% pop concerts (n=16)  29% rock concerts (n=19)  19% rap/hip hop concerts (n=7) |
| Stagelund et al., (24) | 2012 Roskilde Music Festival |  |  |  |  |  | ↑ 238 ED presentations (2.2%) |  |  |
| **Event category: *Policy change*** | | | | | | | | | |
| Atkinson et al., (43) | **Change in trading hours of:** |  |  |  |  |  |  |  |  |
|  | Extending trading to 12am |  |  |  |  |  | ↑ 5.9% ED presentations; margin of error ± 2.6 |  |  |
|  | Extending trading hours to 2am |  |  |  |  |  | ↑ 8.5% ED presentations; margin of error ± 2.3 |  |  |
|  | Extending trading hours to 11pm |  |  |  |  |  | ↑ 5.6% ED presentations; margin of error ±2.7 |  |  |
|  | 1am closing time |  |  |  |  |  | ↓ 19.4% ED presentations; margin of error ±2.3 |  |  |
|  | 3am closing time |  |  |  |  |  | ↓ 11.9% ED presentations; margin of error ±2.1 |  |  |
| Castro-Marin et al., (25) | Alternate Care Site at event |  |  |  |  |  | ↓ 55.3% ED transports (OR=0.37; 95% CI = 0.16-0.86) (p=0.01*) |  |  |
| Fierro-Fine et al., (42) | University alcohol policy | Nongame day (n=3959/5437) | Game day  (n=1478/5437) | ≥ 30  (n=3015/5437) | 63.2% Male (n=3437/5437) |  | ↑ ED presentations post policy change (n=3959/5473)  (p=<0.01*) | ↓ severe ethanol range ≥ 240mg/dL on game days after the implementation of policy |  |
| Fulde et al., (28) | Changes to liquor licencing | ↑ 9.1% ED presentations during high alcohol times (18:00 Friday – 06:00 Sunday) than the rest of the week (3.1%; p < 0.05*) |  |  |  |  | ↓ ED presentations from 318 (pre-implementation) to 246 (post-implementation) (p = <0.05*) |  | Takeaway alcohol sales stop at 22:00; no service of “shots” after midnight; 01:30 – 03:30 “lockout” rule; and 03:00 “last drinks” |
| Gale et al., (38) | Increase in alcopops tax |  |  |  | 66% Male (n=70740/107810) |  | ↓ 18 – 24-year-old female ED presentations (=0.37 presentations/100 000/month, 95%CI -0.45 to -0.29). |  |  |
|  | Introduction of GST |  |  |  |  |  | ↑ ED presentation rates for 18 – 24-year old’s (0.14/100 000/month, 95%CI 0.05 – 0.22) |  |  |
| Grigoletto et al., (27) | COVID-19 Lockdown | ↑ ED presentations post the lock down period (04/05/20 –27/05/20) from 2.96% (n=15) to 11.31% (n=25) | ↓ ED presentations during the lockdown period (10/04/20 – 04/05/20) from 2.95% (n=9) to 0.88% (n=1) | Mean: 16 – 18 (n=9/25) | 68% Male (n=17/25) |  |  | Mean: 240 mg/dL (range: 170 – 320) |  |
| Kharasch et al., (29) | University alcohol policy |  |  | Mean: 19 |  | Mean: 252.5 minutes | ↑ 56% yearly transports to ED following the implementation of the policy (p=<0.01*) | Mean: 236mg/dL (admitted patients)  193mg/dL (non-admitted patients) (p=<0.01*) |  |
| **Disruptive event category: *Public Holiday*** | | | | | | | | | |
| Griffin et al., (35) | All public holidays | More likely to present out of normal working hours (00:00 – 09:00) (p < 0.001*) RR (95% CI):  1.35 (1.20 – 1.51) |  |  |  |  | ↑ 43% of alcohol-related ED presentations on public holidays compared to 38% on all other days (p=<0.001*). RR (95% CI): 1.24 (1.17 – 1.32) |  |  |
|  | Christmas Day |  |  |  |  |  | ↑ 81% risk of alcohol involvement for males. RR (95% CI): 1.81 (1.18-2.76) p=<0.01*  ↑ 79% risk of alcohol involvement for females. RR (95% CI): 1.79 (1.25-2.55) p=<0.01* |  |  |
|  | Christmas Eve |  |  |  |  |  | ↑ 150% risk of alcohol involvement for males. RR (95% CI): 2.51 (1.70-3.73) p=<0.001* |  |  |
|  | Easter Sunday |  |  |  |  |  | ↑ 58% risk of alcohol involvement for females. RR (95% CI): 1.58 (1.17-2.13) p=<0.01* |  |  |
|  | Good Friday |  |  |  |  |  | ↓25 % risk of alcohol involvement for males and females. RR (95% CI): 0.75 (0.60-0.98) p=<0.05* |  |  |
|  | June Bank Holiday |  |  |  |  |  | ↑ 88% risk of alcohol involvement for males. RR (95% CI): 1.88 (1.33-2.65) p=<0.001* |  |  |
|  | New Year’s Day |  |  |  |  |  | ↑ 53% risk of alcohol involvement for males. RR (95% CI): 1.53 (1.15-2.03) p=<0.01*  ↑ 37% risk of alcohol involvement for females. RR (95% CI): 1.37 (1.04-1.81) p=<0.05* |  |  |
|  | New Year’s Eve |  |  |  |  |  | ↑ 102% risk of alcohol involvement for females. RR (95% CI): 2.02 (1.50-2.72) p=<0.001* |  |  |
|  | St. Patricks Day |  |  |  |  |  | ↑ 62% risk of alcohol involvement for females. RR (95% CI): 1.62 (1.23-2.12) p=<0.01* |  |  |
|  | St. Stephens Day |  |  |  |  |  | ↑ 62% risk of alcohol involvement for females. RR (95% CI): 1.62 (1.18-2.22) p=<0.01* |  |  |
| Lloyd et al., (41) | ANZAC Day |  |  |  |  |  | ↑ ED presentations on the day prior to ANZAC day: Β Coeff. 2.73; p=<0.05* |  |  |
|  | New Year’s Day |  |  |  |  |  | ↑ED presentations on the day prior to New Year’s Day: β Coeff. 25.51; p=<0.05* |  |  |
| **Event category: *Social Event*** | | | | | | | | | |
| Callaghan et al., (36) | Hazardous Birthday drinking | Friday and Saturday | Monday, Tuesday and Wednesday | Impact of birthday week on alcohol-related ED presentations/admissions for ages 13 to 30. | For females, significant impact noted in alcohol presentations to ED in the birthday week for 14^th^, 15^th^, 16^th^, 18^th^, 19^th^, 20^th^, 21^st^, 22^nd^, 23^rd^, 24^th^, 25^th^, 26^th^, 29^th^, 30^th^ birthdays.  For males, significant impact noted in alcohol presentations to ED in the birthday week for 16^th^, 17^th^, 18^th^, 19^th^, 20^th^, 21^st^, 22^nd^, 23^rd^, 24^th^, 25^th^, 26^th,^ 30^th^ birthdays. |  | Significant impact noted in alcohol presentations to ED in the birthday week for 14^th^, 16^th^, 17^th^, 18^th^, 19^th^, 20^th^, 21^st^, 22^nd^, 23^rd^, 24^th^, 25^th^, 26^th^, 28^th^, 29^th^, 30^th^ birthdays. Most noticeable for 19^th^ birthday week. Note: in Ontario, Canada minimum legal drinking age is 19 years. |  |  |
|  |  |  | | | | | | | |
| Lloyd et al., (41) | Last working day before Christmas |  |  |  |  |  | ↑ ED presentations on the last working day before Christmas: Β Coeff 2.76 (p=<0.05) |  |  |
| **Event category: *Sporting Event*** | | | | | | | | | |
| Gardener et al., (20) | 2011 Rugby World Cup | Opening ceremony (09/09/11): ↑ ATS 2 and 3 with up to 25 presentations for the hour 20:00 – 20:59  Grand Final (23/10/11 – 24/10/11): sustained peak period between 21:00 – 03:00 |  |  |  |  | ↑8% ED presentations from 6854 to 7419 during event.  44 patients required short-stay observation during the evening of the opening ceremony – nearly threefold increase on the departmental average of 15 per 24 hours. |  | The department saw its highest ever 24hr daily attendances during the two peak periods, resulting in a large number of Ambulances queuing to offload. |
| Noel et al., (19) | EURO-16 Football Cup | Month of June  7.7 (CI 6.5-9)  (p=<0.02*) | Month of August 5.5 (CI 4.7 – 6.5)  (p=<00.2*) | Mean (SD): 44.6 (20.8) | 53.2% Male |  | ↑43% ED presentations on event days (p = 0.003*) |  |  |
| Lloyd et al., (41) | AFL Grand Final |  |  |  |  |  | ↑ ED presentations 2.37, 95% CI: 0.55–4.19 |  |  |
|  | Commonwealth Games |  |  |  |  |  | ↑ ED presentations 2.45, 95% CI: 0.6–4.3 |  |  |
|  | Melbourne Cup Day |  |  |  |  |  | ↑ ambulance attendees 6.14, 95% CI: 2.42–9.85 |  |  |

^Australian Football League (AFL); Australian New Zealand Army Corps (ANZAC); Australasian Triage Score (ATS); Emergency Department (ED); Intensive Care Unit (ICU); Intravenous Fluid (IVF); Confidence Interval (CI)^
